# Mechanical power thresholds during mechanical ventilation: An experimental study

**DOI:** 10.14814/phy2.15225

**Published:** 2022-03-27

**Authors:** Federica Romitti, Mattia Busana, Maria Michela Palumbo, Matteo Bonifazi, Lorenzo Giosa, Francesco Vassalli, Alessandro Gatta, Francesca Collino, Irene Steinberg, Simone Gattarello, Stefano Lazzari, Paola Palermo, Ahmed Nasr, Ann‐Kathrin Gersmann, Annika Richter, Peter Herrmann, Onnen Moerer, Leif Saager, Luigi Camporota, John J. Marini, Michael Quintel, Konrad Meissner, Luciano Gattinoni

**Affiliations:** ^1^ Department of Anesthesiology University Medical Center Göttingen Göttingen Germany; ^2^ Department of Anesthesia and Intensive Care “Ceccarini”Hospital AUSL della Romagna Riccione Italy; ^3^ Department of Anesthesia, Intensive Care and Emergency “Citta’ della Salute e della Scienza” Hospital Turin Italy; ^4^ Department of Pathology Papa Giovanni XXIII Hospital Bergamo Italy; ^5^ Institute of Pathology University Medical Center Göttingen University of Göttingen Germany; ^6^ Outcomes Research Consortium Cleveland Ohio USA; ^7^ Department of Adult Critical Care Guy’s and St Thomas’ NHS Foundation Trust Health Centre for Human and Applied Physiological Sciences London United Kingdom; ^8^ Department of Pulmonary and Critical Care Medicine University of Minnesota and Regions Hospital St. Paul Minnesota USA

**Keywords:** mechanical power, mechanical ventilation, treshold, ventilation induced lung injury

## Abstract

The extent of ventilator‐induced lung injury may be related to the intensity of mechanical ventilation––expressed as mechanical power. In the present study, we investigated whether there is a safe threshold, below which lung damage is absent. Three groups of six healthy pigs (29.5 ± 2.5 kg) were ventilated prone for 48 h at mechanical power of 3, 7, or 12 J/min. Strain never exceeded 1.0. PEEP was set at 4 cmH_2_O. Lung volumes were measured every 12 h; respiratory, hemodynamics, and gas exchange variables every 6. End‐experiment histological findings were compared with a control group of eight pigs which did not undergo mechanical ventilation. Functional residual capacity decreased by 10.4% ± 10.6% and 8.1% ± 12.1% in the 7 J and 12 J groups (*p *= 0.017, *p *< 0.001) but not in the 3 J group (+1.7% ± 17.7%, *p *= 0.941). In 3 J group, lung elastance, PaO_2_ and PaCO_2_ were worse compared to 7 J and 12 J groups (all *p *< 0.001), due to lower ventilation‐perfusion ratio (0.54 ± 0.13, 1.00 ± 0.25, 1.78 ± 0.36 respectively, *p* < 0.001). The lung weight was lower (*p *< 0.001) in the controls (6.56 ± 0.90 g/kg) compared to 3, 7, and 12 J groups (12.9 ± 3.0, 16.5 ± 2.9, and 15.0 ± 4.1 g/kg, respectively). The wet‐to‐dry ratio was 5.38 ± 0.26 in controls, 5.73 ± 0.52 in 3 J, 5.99 ± 0.38 in 7 J, and 6.13 ± 0.59 in 12 J group (*p *= 0.03). Vascular congestion was more extensive in the 7 J and 12 J compared to 3 J and control groups. Mechanical ventilation (with anesthesia/paralysis) increase lung weight, and worsen lung histology, regardless of the mechanical power. Ventilating at 3 J/min led to better anatomical variables than at 7 and 12 J/min but worsened the physiological values.

## Graphical abstract

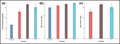

## 1 INTRODUCTION

The energy load transferred to the lung during mechanical ventilation generates mechanical stress and strain, and this load is believed causal to ventilator‐induced lung injury (VILI)(Mead et al., 1970). When the respiratory rate is taken into account together with inflation pressure and flow, a summary variable may be computed and defined as “mechanical power”, which represents the total energy transferred to the respiratory system per unit of time (Gattinoni et al., 2016).

Therefore, it seems clinically important to assess whether a mechanical power threshold exists below which mechanical ventilation may be considered completely “safe”. Clinical observational data in ARDS suggest that a value of respiratory system (Power_RS_) greater than 17 J/min is associated with more frequent adverse events (Sepra, 2018). In the present study we aimed to determine the existence of an absolute threshold value of mechanical power, below which the lung remains uninjured throughout the 48‐h experimental model of mechanical ventilation during general anesthesia and muscle relaxation.

## 2 METHODS

The study population consisted of 18 female domestic pigs (body weight 29.5 ± 2.5 kg) handled according to the Helsinki Declaration (LAVES Antragsnummer 18/2795). All animals were endotracheally intubated and ventilated prone (the natural decubitus of the animal) in volume‐controlled mode and maintained sedated and paralyzed throughout the experiment. Arterial line for PiCCO^®^ measurements, esophageal balloon, central venous, pulmonary artery, and urinary catheters were placed. Fluid balance was maintained by an infusion of balanced crystalloids (1 ml/h) and general anesthesia by continuous infusions of propofol, midazolam, and sufentanil. Further eight healthy pigs (weight 40.6 ± 7.9 kg) sacrificed immediately after anesthesia induction (i.e., without receiving mechanical ventilation), served as healthy controls for the end‐experimental variables.

### 2.1 Experimental design

Mechanical power was calculated as:

$$MP=0.098\times TV\times RR\times \left[Paw_{\mathrm{peak}}‐\frac{1}{2}\left(Paw_{\mathrm{plat}}‐Paw_{\mathrm{ee}}\right)\right]$$

where *TV* is the tidal volume in liters, *RR* is the respiratory rate in breaths per minute, *Paw_peak_* is the peak inspiratory pressure, *Paw_plat_* is the plateau pressure, and *Paw_ee_* is the end‐expiratory pressure in cmH_2_O.

The initial setting of the ventilator was FiO_2_ = 0.4, PEEP = 4 cmH_2_O, TV = 8 ml/kg, Respiratory rate = 18 bpm to provide 7 J/min. The mechanical power was successively randomly raised to 12 J/min or decreased to 3 J/min to constitute three experimental groups (3 J, 7 J, and 12 J, six animals each) by adjusting TV and respiratory rate (each by 10% steps). The other variables were kept unchanged. The experiment last 48 h and respiratory mechanics, gas exchange, and hemodynamic variables were collected at baseline (h 0), after 30 min from reaching the target mechanical power (h 0.5) and, subsequently, every 6 h.

### 2.2 End‐experiment

The animals were sacrificed with a bolus of thiopental (4 g) and potassium chloride (40 mEq). Samples for wet‐to‐dry analysis were obtained from the apical, middle, and basal region of both lungs, as well as from liver, kidney, small bowel, and muscle (2 g/piece oven‐dried for 24 h at 50°C). The lungs were preserved and stored for subsequent histological examination. Twenty samples for each animal were acquired (five ventral and five dorsal per lung, along the cranio‐caudal axis), and they were analyzed for the following variables: alveolar edema, vascular congestion, perivascular edema, septal ruptures, inflammation, atelectasis, intravascular thrombi, hyaline membranes, and intra‐alveolar hemorrhages. For further details, please consult the Online Supplement (https://doi.org/10.6084/m9.figshare.16611724).

### 2.3 Statistical analysis

The sample size was not formally a priori calculated due to the explorative nature of the study and was decided according to our previous experience. Data are presented as means ± standard deviation or median [interquartile range] as appropriate, depending on the data distribution. Baseline and end‐experiment differences as between‐subjects factors across 3 J, 7 J, or 12 J groups were assessed with one‐way analysis of variance. The strength of the relationship was tested with linear regression. To evaluate the effect of time and to account for the repeated measures design, we used a linear mixed effects model with mechanical power group, time, and their interactions as fixed effects, and the animal subject as the random effect. *Post hoc* analyses were adjusted with Tukey's correction. Two tailed *p*‐values <0.05 were considered statistically significant. All analyses were performed with R for Statistical Computing 4.0.

## 3 RESULTS

In Table 1 we summarize the most relevant physiological variables recorded during the baseline ventilation period before applying the experimental ventilatory setting (h 0) for each experimental group. As shown, the baseline variables were not statistically different among the experimental groups, with the exception of significantly higher elastances and associated variables in the 7 J group. In Table 2 we present the ventilatory settings targeted to reach 3, 7, or 12 J/min of mechanical power. The actual mechanical power delivered to each group was 2.91 ± 0.18 J/min, 7.38 ± 0.67 J/min, and 11.70 ± 0.76 J/min (*p *< 0.001). These values remained unmodified for each animal throughout the experiment (see Figure S1).

**TABLE 1:** Anatomic, respiratory, mechanichal, gas exchange and hemodynamic variables recorded at base line in the three experimental groups

	3 J	7 J	12 J	*p* value
Anatomical characteristics				
Weight (kg)	29.0 ± 3.0	29.2 ± 2.4	30.3 ± 2.2	0.648
Functional Residual Capacity (ml)	421 ± 61	426 ± 75	425 ± 76	0.998
FRC/kg	14.6 ± 2.5	14.6 ± 1.8	14.0 ± 1.8	0.836
Respiratory mechanics				
Tidal volume (ml)	270 ± 14	281 ± 20	271 ± 18	0.502
Respiratory rate (bpm)	21.3 ± 0.8	20.8 ± 1.5	21.0 ± 1.5	0.803
Plateau pressure (cmH_2_O)	12.4 ± 0.7	14.9 ± 1.1	13.2 ± 0.8	<0.001
Driving pressure (cmH_2_O)	8.2 ± 0.7	10.6 ± 1.2	9.2 ± 0.9	<0.001
End‐Expiratory pressure (cmH_2_O)	4.2 ± 0.2	4.2 ± 0.2	4.1 ± 0.2	0.376
Elastance_RS_ (cmH_2_O/L)	30.5 ± 3.7	37.8 ± 3.2	34.0 ± 4.9	0.019
Elastance_lung_ (cmH_2_O/L)	19.3 ± 5.7	22.9 ± 3.4	18.1 ± 3.6	0.171
Elastance_cw_ (cmH_2_O/L)	11.2 ± 3.3	14.9 ± 3.5	15.9 ± 2.4	0.042
Stress (cmH_2_O)	7.8 ± 1.8	9.0 ± 1.0	7.0 ± 0.8	0.051
Strain	0.75 ± 0.1	0.74 ± 0.1	0.73 ± 0.1	0.729
Mechanical power_RS_ (J/min)	6.7 ± 0.4	7.5 ± 0.6	6.7 ± 0.3	0.012
Gas exchange				
PaO_2_ (mmHg)	201.0 ± 19.9	203.1 ± 23.8	210.3 ± 19.7	0.778
PaO_2_/FiO_2_ (mmHg)	503 ± 50	507 ± 60	524 ± 49	0.778
Minute ventilation (L/min)	5.92 ± 0.23	5.98 ± 0.20	5.70 ± 0.25	0.119
PaCO_2_ (mmHg)	46.7 ± 4.1	48.8 ± 7.7	48.0 ± 4.0	0.792
EtCO_2_ (mmHg)	43.4 ± 5.8	42.8 ± 6.5	43.0 ± 6.9	0.989
Dead space (%)	48.2 ± 4.8	51.0 ± 3.5	47.8 ± 4.6	0.405
Alveolar ventilation (L/min)	3.05 ± 0.32	2.85 ± 0.33	2.97 ± 0.27	0.387
VO_2_ (ml/min)	148 ± 22	174 ± 73	146 ± 16	0.590
VCO_2_ (ml/min)	192 ± 20	186 ± 27	196 ± 24	0.761
Hemodynamics				
Mean arterial pressure (mmHg)	92.0 ± 10.3	97.7 ± 9.9	100.0 ± 14.6	0.464
Heart rate (bpm)	82 ± 7	95 ± 27	94 ± 17	0.389
Cardiac output (L/min)	3.46 ± 0.93	3.10 ± 0.57	2.97 ± 0.68	0.512
Mean pulmonary pressure (mmHg)	17.7 ± 6.3	24.2 ± 4.9	24.2 ± 8.6	0.211
Wedge pressure (mmHg)	6.3 ± 1.9	4.8 ± 1.1	6.7 ± 2.3	0.272
PvO_2_ (mmHg)	43.0 ± 4.9	39.2 ± 7.5	42.8 ± 4.4	0.443
SvO_2_ (%)	69.6 ± 7.4	63.2 ± 17.8	67.0 ± 8.8	0.662
Hemoglobin (g/dl)	10.3 ± 0.4	9.8 ± 0.5	10.0 ± 0.5	0.104

**TABLE 2:** Ventilatory setting applied in the three experimental groups to reach their mechanical power target of 3, 7, and 12

	3 J	7 J	12 J	*p* value
Tidal volume (ml)	201, 16	281, 19	347.0, 8.7	<0.001
Tidal volume/kg (ml/kg)	6.9, 0.3	9.7, 0.7	11.5, 1.1	<0.001
Respiratory rate (bpm)	15.7, 1.0	20.7, 1.4	23.3, 1.5	<0.001
Inspiratory time (sec)	1.23, 0.17	0.97, 0.07	0.88, 0.07	<0.001
Inspiratory flow (L/min)	12.2, 3.98	17.3, 1.11	23.2, 2.51	<0.001
PEEP (cmH_2_O)	3.9, 0.2	4.0, 0.3	4.3, 0.3	0.025
Mechanical power (J/min)	2.91, 0.18	7.38, 0.67	11.7, 0.76	<0.001

### 3.1 Physiological variables

#### 3.1.1 Respiratory mechanics and lung volumes

The time course of mean airway pressure, end‐expiratory pressure, plateau pressure, and peak pressure is shown in Figure S2–S5. The measured end‐expiratory pressure averaged 3.98 ± 0.20 (3 J), 4.26 ± 0.27 (7 J), and 4.41 ± 0.27 cmH_2_O (12 J); *p *< 0.001), despite a set PEEP of 4 cmH_2_O, indicating the presence of auto (intrinsic) PEEP that was proportional to the mechanical power applied.

**FIGURE 1:**
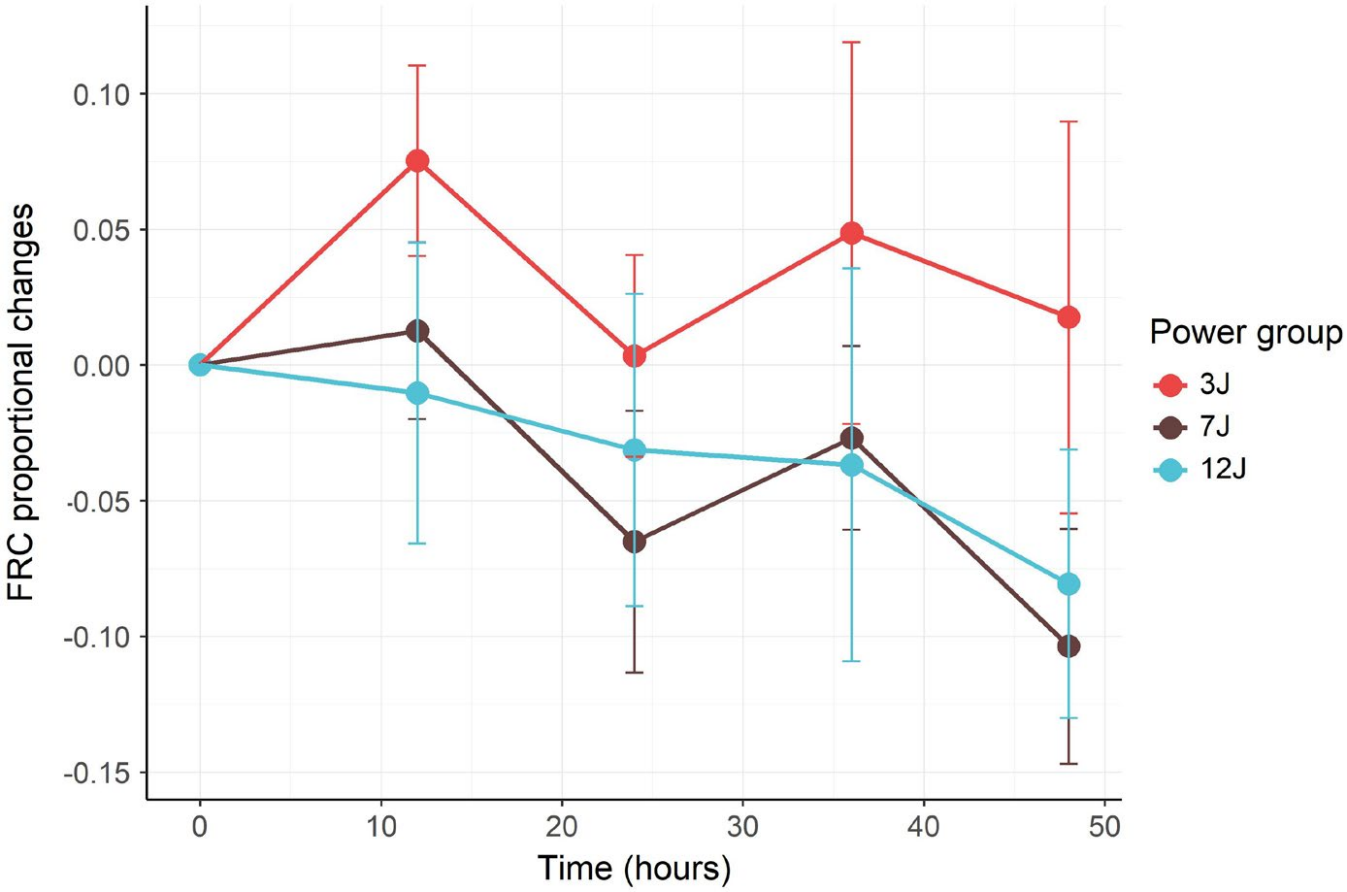
Time course of FRC *changes* (actual FRC – baseline FRC/baseline FRC) as a function of time in groups 3 J, 7 J, and 12 J. The baseline FRC were 421, 426, and 425 ml respectively, see also Table 2 (*p* value for time = 0.025, power group = 0.932, time and power group interaction = 0.222). The error bars represent the standard error of the mean

Compared to baseline, FRC at the end of the experiment remained constant in the 3 J group (1.7% ± 17.7%, *p *= 0.941), while decreased (indicating a reduction in the lung gas content) with higher mechanical power (10.4% ± 10.6% in the 3 J group and 8.1% ± 12.1% in the 12 J groups, respectively, *p *= 0.017 and *p *= 0.07, Figure 1).

#### 3.1.2 Hemodynamics

The cardiac output was highest in the group receiving the lowest mechanical power (Figure S6). The mean arterial pressure, pulmonary artery pressure, and central venous pressure significantly decreased over time without differences across the three groups, as shown in Figures S7–S9.

#### 3.1.3 Gas exchange

**FIGURE 2:**
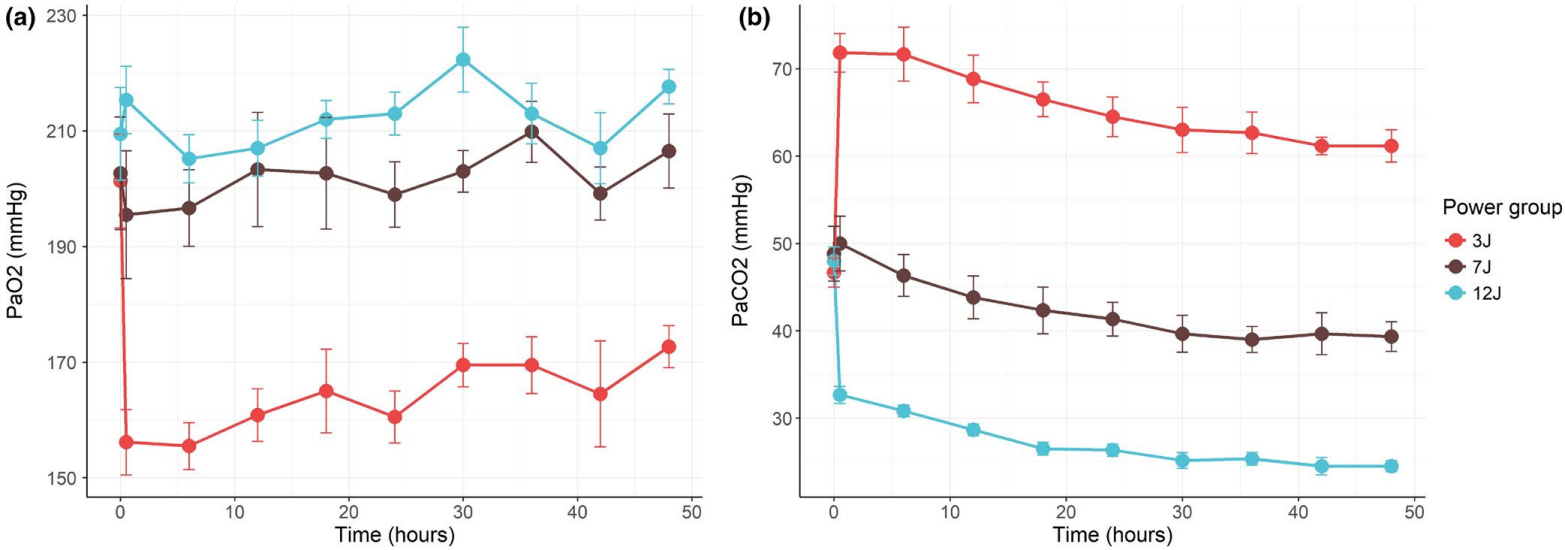
PaO_2_ (panel a) and PaCO_2_ (panel b) as a function of time in groups 3 J, 7 J, and 12 J. The FiO_2_ was maintained constant at 0.4 in all animals throughout the whole experiment (PaO_2_: *p* value for time = 0.251, power group: <0.001, time and power group interaction = 0.368; PaCO_2_: time = <0.001, power group = <0.001, time and power group interaction = <0.001). The error bars represent the standard error of the mean

**FIGURE 3:**
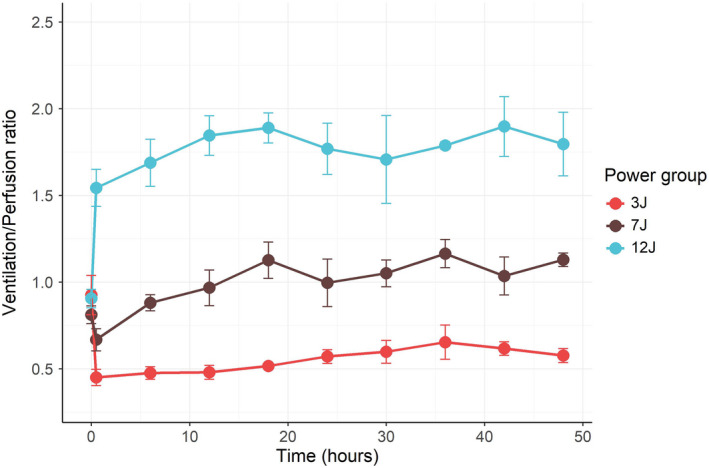
Alveolar ventilation to perfusion ratio (V_A_/Q) as a function of time in groups 3 J, 7 J, and 12 J (*p* value for time = 0.737, power group = <0.001, time and power group interaction = 0.349). Eight out of 180 measurements missing. The error bars represent the standard error of the mean

As shown in Figure 2, the PaO_2_ was lower and PaCO_2_ was higher in the 3 J group, compared to groups 7 J and 12 J, indicating worse gas exchange. Venous admixture was significantly higher in the 3 J group (Figure S10). The anatomical dead space was significantly higher in the 3 J group compared to the 7 J and 12 J groups (Figure S11). The global alveolar ventilation/perfusion (V_A_/Q) ratio was strikingly different among the three groups throughout all the experiments (Figure 3) and increased proportionally to the mechanical power applied.

#### 3.1.4 End‐experimental variables

**FIGURE 4:**
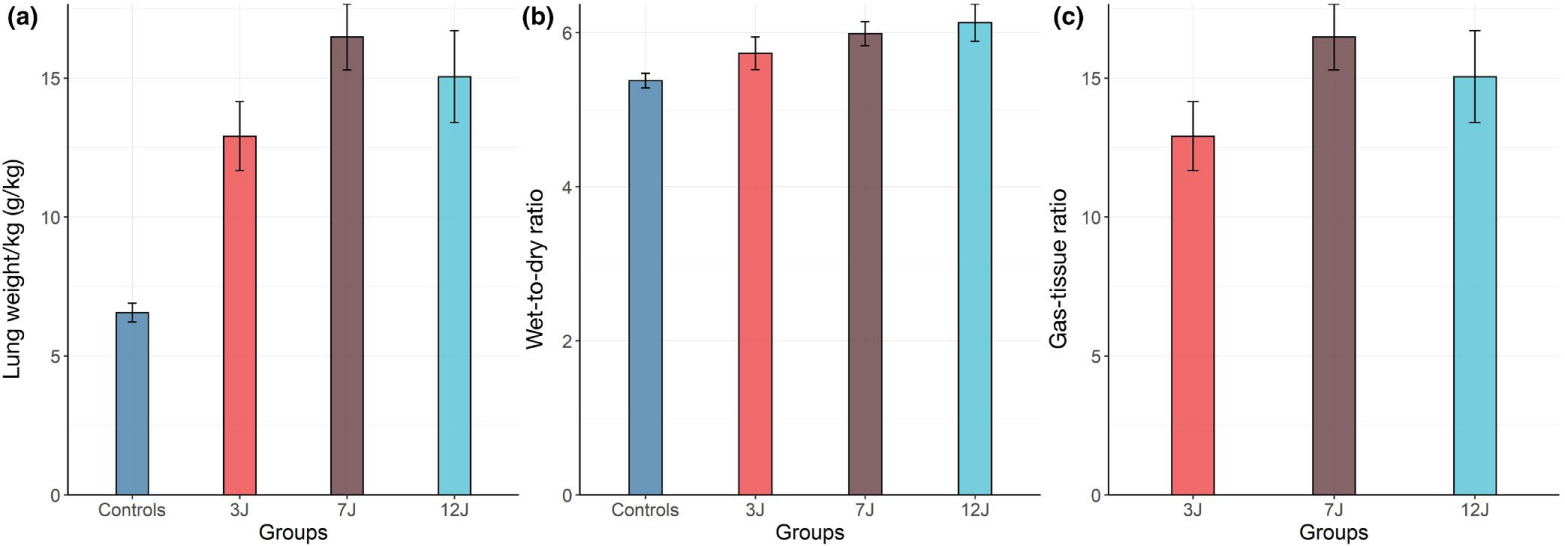
End‐experimental lung weight/kg, lung wet‐to‐dry ratio, and gas/tissue ratio in the experimental groups. The gas/tissue ratio was not available in the control animals, as FRC was not measured (*p* values: lung weight/kg: group: <0.001; lung wet‐to‐dry ratio: group: 0.033; gas/tissue ratio: 0.097). The error bars represent the standard error of the mean

After the 48 h of the experiment, the fluid balance was not different among the three groups, averaging +615 ± 539 ml, +357 ± 731 ml, and +766 ± 386 ml in the 3, 7, and 12 J groups, respectively (*p *= 0.470). The lung weight/kg, the wet‐to‐dry ratio, and the gas‐to‐tissue ratio measured in the non‐mechanically ventilated controls and in the 3 J, 7 J, and 12 J mechanically ventilated animals are presented in Figure 4. As shown, lung weight and wet‐to‐dry ratio were significantly different for controls and the 3 J, 7 J, and 12 J groups, but did not reach the statistical significance within the three groups of mechanically ventilated animals.

**FIGURE 5:**
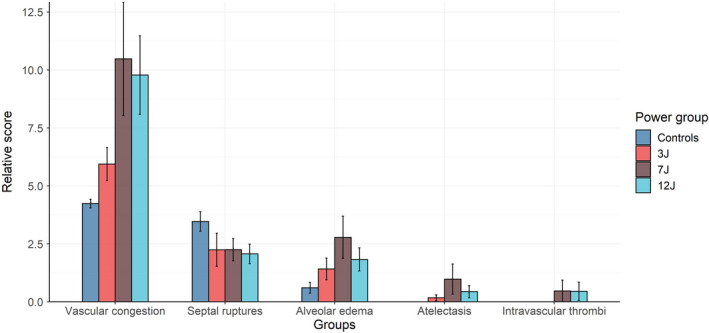
Arbitrary score of the histological alterations observed in the experimental groups (vascular congestion: *p* value for power group = 0.010; septal ruptures: *p* value for power group = 0.175; alveolar edema: *p* value for power group = 0.054; atelectasis: *p* value for power group = 0.187; intravascular thrombi: *p* value for power group = 0.459). The error bars represent the standard error of the mean

The histological findings are summarized in Figure 5. As shown, vascular congestion was the most frequent histological alteration, significantly more severe in 7 J and 12 J compared to controls and 3 J, and examples of histological preparations can be observed in Figure S12.

## 4 DISCUSSION

The main findings of this study are as follows: (a) Compared to controls, even a mechanical power as low as 3 J/min caused an increase in the lung weight and lung wet‐to‐dry ratio, and worsening of histological parameters of lung injury; (b) These physiological parameters worsened with the increase in mechanical power to 7 J/min and 12 J/min; (c) However, gas exchange‐related variables were significantly worse in the 3 J group than in the 7 J and 12 J groups. These results describe that mechanical ventilation per se (with all the elements necessary to maintain mechanical ventilation, e.g., sedation, paralysis etc.) increases lung injury, and this injury then is proportional to the intensity of mechanical ventilation. No safe threshold could be identified that guarantees safe lung protective ventilation.

### 4.1 Lung mechanics

In this study we applied a low‐rate sigh (Bendixen et al., 1963) and 4 cmH_2_O PEEP (Collino et al., 2019) to counteract the effects of anesthesia and paralysis on FRC which, nonetheless, was significantly reduced in the 7 J and 12 J groups, compared to their baseline values. The latter groups were characterized by strain levels that were _~_50% and _~_100% greater than in 3 J group, respectively (Figure S13). However, even the highest strain reached in 12 J group (strain _~_1) was remarkably less than the threshold of 1.5 needed to cause noteworthy edema in our previous work (Cressoni et al., 2016; Protti et al., 2011; Vassalli et al., 2020). Despite this, these lower strain values were associated with modest but significant decrease in lung volumes of approximately 20% in groups 7 J and 12 J, compared to baseline. The lower lung volumes were accompanied by higher lung elastance and lung weights in 7 J and 12 J, compared to the 3 J group (although not statististcally different) suggesting the presence of tissue edema and compression atelectasis in the former (Pelosi et al., 1994). These data support the concept that it is not only the strain per cycle alone that determines VILI, but also the mechanical power applied. Power includes not only strain, which is tidal volume dependent, but also respiratory rate and PEEP (Gattinoni et al., 2016).

### 4.2 Gas exchange

#### 4.2.1 Oxygenation

The groups 7 and 12 J, despite higher lung elastance and lower lung volumes, which indicate the presence of higher amount of atelectasis, had better oxygenation than group 3 J/min, the opposite of what one may anticipate. However, we previously found in a similar experimental model of VILI that oxygenation was not impaired, even in presence of considerable atelectasis, presumably because unstable units reopen easily during inspiration, preserving near‐normal oxygenation (Cressoni et al., 2016). This may explain nearly normal oxygenation in groups 7 J and 12 J, but it does not explain the decreased oxygenation in 3 J group, where the amount of atelectasis was likely negligible (maintained gas volume, use of PEEP and sigh). Therefore, the relatively worse oxygenation in this group is likely attributable to a remarkably lower V_A_/Q rather than true shunt. Indeed, the alveolar ventilation was about 1/3 lower in the 3 J compared to 7 J and 12 J groups, while the cardiac output was about 1/3 higher, resulting in a calculated V_A_/Q well below 1.

#### 4.2.2 CO_2_ clearance

The comparatively large differences of PaCO_2_ across groups are easily explained by their different levels of alveolar ventilation. Indeed, the anatomical dead space, which includes a large apparatus dead space, was larger in the 3 J group compared to 7 J and 12 J animals, due to different tidal volumes in the three groups, despite similar total CO_2_ production among them. The arterial pH followed the PaCO_2_ level.

#### 4.2.3 Hemodynamics

Applying 3, 7, or 12 J /min mechanical power immediately modified the hemodynamics. Indeed, the two higher levels of mechanical power were associated with greater inspiratory and expiratory (due to a slight auto‐PEEP) airway and pleural pressures. These variables were linearly and inversely related to the cardiac output. Indeed, in the 3 J group the cardiac output was almost 30% higher than those measured in the 7 J and 12 J groups. However, beyond the airway pressures, the effects of the PaCO_2_ level on cardiac output cannot be ignored. To maintain oxygen consumption (similar in the three groups), decreases in cardiac output were associated with progressive increases of oxygen extraction, as documented by the decreases of SvO_2_ and consequent increases of (a‐v)O_2_ difference (Figures S14 and S15). To maintain adequate systemic and pulmonary pressures despite a decrease in cardiac output, the peripheral and pulmonary resistances progressively increased from group 3 J to 12 J.

### 4.3 End––experimental variables

#### 4.3.1 Anatomical variables

The group of animals that did not undergo mechanical ventilation averaged lower lung weight and wet‐to‐dry ratio, when compared to the 3 J group. These discrepancies indicate that we were unable to maintain the lung free of edema when undergoing 48 h of mechanical ventilation, even at low power of 3 J/min. Multiple theoretical reasons may account for these findings. Most prominent among these possibilities are the hemodynamic/water balance consequences of applying positive instead of physiological negative intrathoracic pressure (Cournand & Motley, 1948; Demling et al., 1975; Grasso et al., 2008), the altered distribution of tidal volume due to the diaphragm paralysis (Froese & Bryan, 1974), the monotony of the ventilating pattern as compared to spontaneous breathing (Putensen et al., 1999), impaired pulmonary lymphatic drainage (Frostell et al., 1987), the complete loss of muscle tone due to neuromuscular agents, and the absolute immobility (Ray et al., 1974) maintained for 48 h. However, despite these shared features of the three mechanically ventilated groups of animals, the 3 J group showed lower values of lung weight and wet‐to‐dry ratio than the 7 and 12 J groups. These variables tend to increase with mechanical power, as also shown in our previous works in which we used mechanical power levels remarkably greater than the ones used in the 12 J group (Collino et al., 2019; Cressoni et al., 2016). In the present experiment the wet‐to‐dry ratio of the liver was significantly higher in 7 J and 12 J group compared to 3 J, as shown in the Online Supplement.

#### 4.3.2 Histology

The most frequent histological abnormality was vascular congestion, while such alterations of the lung structure per se, such as septal ruptures or emphysema‐like lesions were less frequent. In this study we never observed a lung hepatization patterns with complete loss of lung structure that we observed in previous studies, where much higher levels of mechanical power were used (Vassalli et al., 2020). Nevertheless, even the lungs of the animals treated with 3 J/min of mechanical power showed histological alterations when compared to the controls.

#### 4.3.3 Mechanical power and threshold

In this study we show that ventilation per se, at least when associated to deep sedation and paralysis,–regardless of the mechanical power–causes lung injury and we were not able to identify any mechanical power threshold below which we were able to prevent VILI, even at transpulmonary mechanical power levels similar to what is expected in spontaneously breathing pigs of similar size (1–1.5 J/min). Given that the maintenance of anatomical normality after 48 h of mechanical ventilation was, at least in our hands, not achievable, this opens the discussion what might be considered an acceptable threshold when positive pressure ventilation is applied, balancing between the consecutive anatomical and physiological alterations. If we consider the bulk of data related to gross anatomy and physiology, there is clear indication that the 3 J is better than 7 J. Unfortunately, this level of mechanical power was associated with moderate‐to‐severe impairment of key physiological variables. At 7 J/min, the physiological variables were distinctly better, while the anatomical variables were slightly worse. At 12 J/min both anatomical and physiological variables were more compromised. This study has several limitations: for the sake of simplicity, we referred to the mechanical power applied to the whole respiratory system, instead of the transpulmonary mechanical power applied directly to the lung parenchyma itself. In addition, it is difficult to directly translate our experimental data in pigs to the human being. Indeed, several differences should be taken into account: the lung volumes are sharply different, requiring appropriate normalization. More importantly, the specific elastance of the pig's lung (_~_6–8 cmH_2_O) is less than that the human being (_~_12–14 cmH_2_O), suggesting that a similar power, theoretically, induces a comparatively less severe damage in humans (Chiumello et al., 2008; Protti et al., 2011). In addition, we used healthy animals to study VILI. The advantage is to avoid confounding variables; the disadvantage is that we ignore the consequences of the mechanical power distribution in an inhomogeneous lung.

## 5 CONCLUSION

Mechanical ventilation is associated with lung injury even at low mechanical power. The best compromise between severe histological injury and gas exchange was obtained at *respiratory system* mechanical powers between 3 and 7 J/min. This strongly suggests that maintenance of acceptable gas exchange during mechanical ventilation can only be achieved at the price of higher mechanical power and worse lung injury. This should be taken into account when “ultraprotective approach” is considered (Hager et al., 2005).

## CONFLICT OF INTEREST

LG reports to be consultant for General Electrics and SIDAM. He also receives lectures fees from Estor and Dimar. LS reports financial relationships with Medtronic, Ferrer Deutschland and Merck. Part of the salary support for the author M.B. was provided by an unrestricted research grant from Sartorius Inc. Göttingen, Germany. All other authors disclose no conflict of interest.

## Supporting information



Supplementary Material
