# Supplementary material for: Mechanical power thresholds during mechanical ventilation: An experimental study
Source: Physiol Rep. 2022 Mar 27;10(6):e15225. doi: 10.14814/phy2.15225 (PMC8957661; doi:10.14814/phy2.15225)
Supplement: Supplementary file 1 — Supplementary Material [file PHY2-10-e15225-s001.docx]

## Mechanical power thresholds during mechanical ventilation: an experimental study

### SUPPLEMENTARY MATERIAL

Federica Romitti^1^, M.D.*, Mattia Busana^1^ M.D.*, Maria Michela Palumbo^1^ M.D., Matteo Bonifazi^1^ M.D., Lorenzo Giosa^1^ M.D., Francesco Vassalli^1^ M.D., Alessandro Gatta^2^ M.D., Francesca Collino^3^ M.D., Irene Steinberg^1^ M.D., Simone Gattarello^1^ M.D., Stefano Lazzari^1^ M.D., Paola Palermo^1^ M.D., Ahmed Nasr^4^ M.D., Ann-Kathrin Gersmann^5^ M.D., Annika Richter ^5^ M.D., Peter Herrmann^1^ MSc., Onnen Moerer^1^ M.D., Leif Saager^1,6^ M.D., Luigi Camporota^7^ M.D., John J. Marini^8^ M.D., Michael Quintel^1^ M.D., Konrad Meissner^1^ M.D., Luciano Gattinoni^1^ M.D., FRCP

## Supplementary methods

### Animal preparation

The animals were instrumented with the following devices:

- Endotracheal tube (size 6.5/7 mm)
- Urinary catheter (5 Fr)
- Adult esophageal catheter (8 Fr) Smartcath with esophageal balloon; the correct positioning of the esophageal catheter was checked by an end-expiratory occlusion test
- Central venous catheter (5 Fr) in the jugular vein, ultrasound-guided
- Swan-Ganz catheter (5 Fr) through an introducer (7 Fr) in the jugular vein, ultrasound guided
- Arterial PiCCO catheter (5 Fr) in the femoral artery, ultrasound guided

Temperature management was achieved with a thermic blanket. The aimed temperature was between 38 and 39 °C throughout the experiment, to account for the higher basal temperature of the pig compared to the human being.

### Fluid management

Maintenance: 1 ml/h of Stereofundin 1/1. Additional fluids were administered in form of Stereofundin 1/1 load of maximum 250 ml in 5-10 min if:

- Hemodynamic instability (MAP < 60 mmHg)
- Signs of hypoperfusion (raised lactates, skin mottling, decreased urine output) **and** dynamic preload indices:
- Pulse pressure variation (PPV) > 13%
- Systolic volume variation (SVV) > 12%

We started noradrenaline if the animal was not responsive to the fluid challenge. Colloids were solely administered in case of emergent hemorrhage (i.e., following cannulation). The fluid balance was calculated as total fluid intake from every source (infusions, cardiac output boluses etc…) minus the total fluid lost (urine, gastric content, blood samples) at each time interval. We checked the fluid balance every 6 hours using the calculator we on-purpose prepared.

### Anesthesia administration

General anesthesia was maintained in accordance with local guidelines by continuous infusions of propofol (5.45 ± 1.05 mg · kg−1 · h−1), midazolam (0.93 ± 0.22 mg · kg−1 · h−1) and sufentanil (1.88 ± 0.46 µg · kg−1 · h−1). A low-rate sigh (1 each 100 breaths, 1.5 x tidal volume) was applied to counteract the effects of anesthesia and paralysis on Functional Residual Capacity (FRC).

### Measurements

At each time point, we measured: arterial and mixed venous blood gases, hemodynamics, and volumetric capnography. Respiratory mechanics were measured both at PEEP and at ZEEP after airway suctioning and a recruitment maneuver (Pressure-controlled ventilation, maintained for 30 seconds, with inspiratory pressure of 35 cmH_2_O, and 3 seconds of inspiratory time). The Functional Residual Capacity (FRC), was measured at atmospheric pressure, every 12 hours by the helium dilution technique. The strain was calculated as the ratio between tidal volume+PEEP volume and FRC. The alveolar ventilation to perfusion (V_A_/Q) ratio was computed as the ratio between the measured alveolar ventilation to the measured cardiac output. A full list of the formulas for calculated variables can be found in the Online Supplement.

### Calculated variables

The following variables were calculated:

Respiratory system driving pressure (cmH_2_O) = Paw_plat_ – Paw_esp_

Transpulmonary driving pressure (cmH_2_O) = (Paw_plat_ – Paw_esp_) – (Pes_plat_ – Pes_esp_)

Respiratory system elastance (cmH_2_O/l) = (Paw_plat_ – Paw_esp_) / Tidal volume

Chest wall elastance (cmH_2_O/l) = (Pes_plat_ – Pes_esp_) / tidal volume

Lung elastance (cmH_2_O/l) = [(Paw_plat_ – Paw_esp_) – (Pes_plat_ – Pes_esp_)] / Tidal volume

Strain = (Tidal volume + PEEP volume) / FRC (PEEP volume determined by a PEEP release maneuvre, where the expired volume is measured when going from PEEP to ZEEP). FRC was measured with the helium dilution method.

Stress (cmH_2_O) = (Paw_plat_ – Paw_ZEEP_) – (Pes_plat_ – Pes_ZEEP_) (Pes was measured thanks to the esophageal ballon placed at the beginning of the experiment).

Specific lung elastance (cmH_2_O) = stress / strain

Venous admixture = $\frac{{CcO}_{2}-{CaO}_{2}}{{CcO}_{2}-{CvO}_{2}}$

Where CcO_2_ is the end-capillary O_2_ content and is calculated as follows:

$$C_{c}O_{2}=1.39 \cdot\left[ Hb \right]\cdot+0.003\cdot PAO_{2}$$

Where [Hb] is the hemoglobin concentration in g/dl, and PAO_2_ is the alveolar partial pressure of oxygen.

CaO_2_ is the arterial O_2_ content and is calculated as follows:

$$C_{a}O_{2}=1.39 \cdot\left[ Hb \right]\cdot\left[ SaO_{2} \right]+0.003\cdot{PaO}_{2}$$

Where SaO_2_ is the arterial oxygen hemoglobin saturation fraction and PaO_2_ is the arterial partial pressure of oxygen.

CvO_2_ is the mixed venous O_2_ content and is calculated as follows:

$$C_{v}O_{2}=1.39 \cdot\left[ Hb \right]\cdot\left[ SvO_{2} \right]+0.003\cdot{PvO}_{2}$$

Where SvO_2_ is the mixed venous oxygen hemoglobin saturation fraction and PvO_2_ is the mixed venous partial pressure of oxygen.

The artero-venous (a-v) O_2_ difference was calculated as:

$$\left( a-v \right)O_{2}={C_{a}O}_{2}-C_{cv}O_{2}$$

Physiological dead space fraction = $\frac{{PaCO}_{2}- {PECO}_{2}}{{PaCO}_{2}}$

Where PaCO_2_ is the arterial CO_2_ pressure (mmHg), as obtained by the blood gas analyzer, and PECO_2_ is the mean expiratory CO_2_ (mmHg), as measured by the ventilator.

Mechanical Power

$${Power}_{rs}=0.098\cdot RR\cdot\Delta V\cdot{(P}_{peak}-\frac{1}{2}\cdot\Delta P)$$

### Histology

The lungs were fixed in 4% formalin for at least one week. Twenty samples of lung tissue were obtained from each animal for histological examination: for each lung, 10 samples were obtained and each one further divided into a dorsal and ventral portion. The samples were fixed, dehydrated in a graded alcohol series, cleared in xylene and embedded in paraffin. Two µm sections were cut with a microtome, mounted on slides, and stained with hematoxylin-eosin for histological analysis. The sections were viewed under a light microscope, and histological scoring was performed by an investigator blinded to the group allocation of the animals. Scoring was attributed with the following methodology: for each one of the variables of interest (vascular congestion, perivascular edema, septal ruptures, inflammation, atelectasis, intravascular thrombi, hyaline membranes and intra-alveolar hemorrhages) the scores of absent (0-25% of the optical field, 2 points), focal (25-50% of the optical field, 4 points), multifocal (50-75% of the optical field, 8 points) and diffuse (75-100% of the optical field, 16 points) were attributed. “Apical” were considered the samples 1 to 4 (right lung) and 11 to 14 (left lung). “Basal” were considered the samples 7 to 10 (right lung) and 17 to 20 (left lung). The final score used in the analysis is the average of the scores for each variable.

## Supplementary results

The error bars in all figures represent the standard error of the mean.


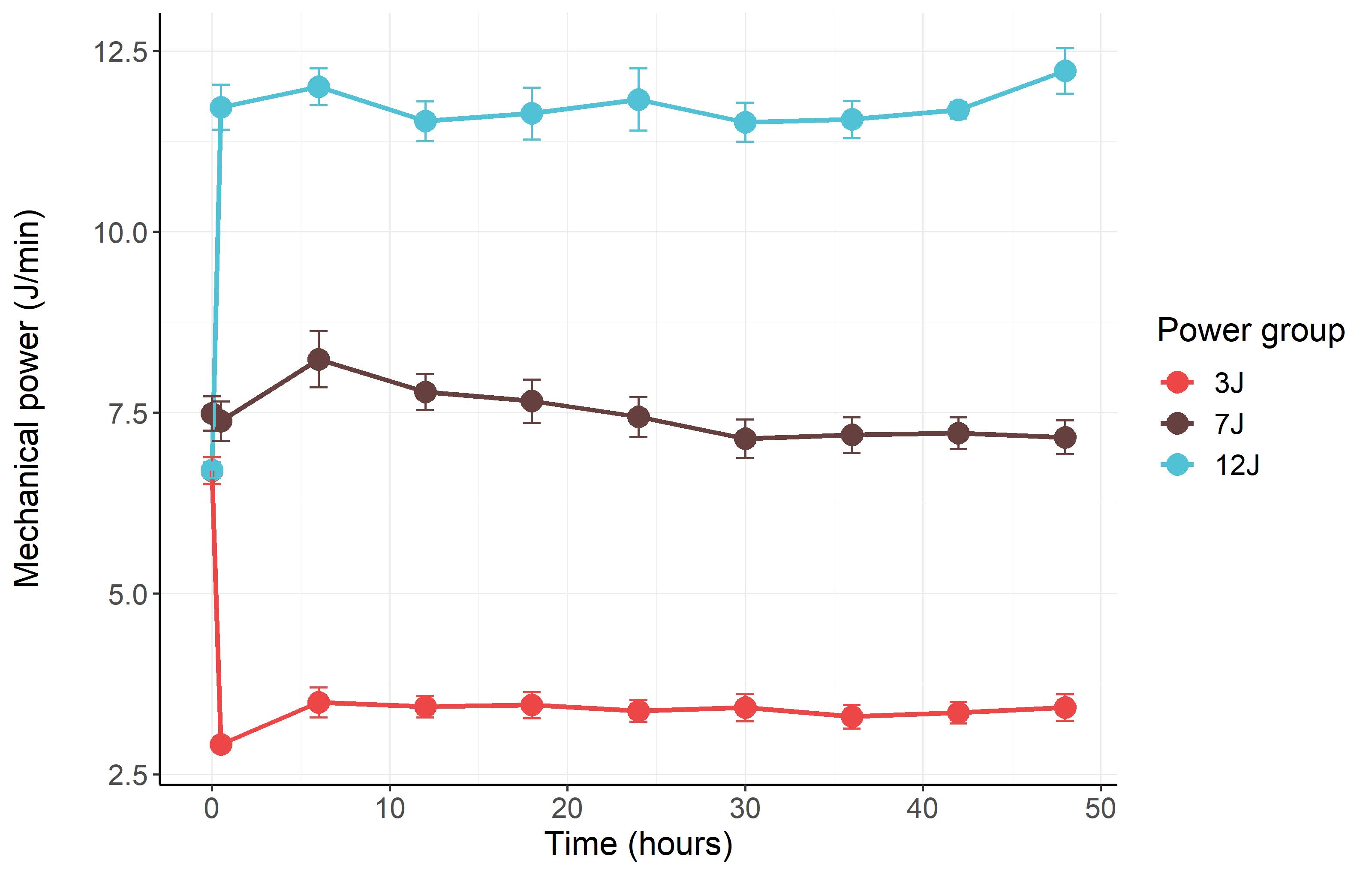


Figure E1: Total respiratory system mechanical power as a function of time in groups 3J, 7J and 12J (p values: time = 0.69, power group = < 0.001, time and power group interaction = < 0.001).


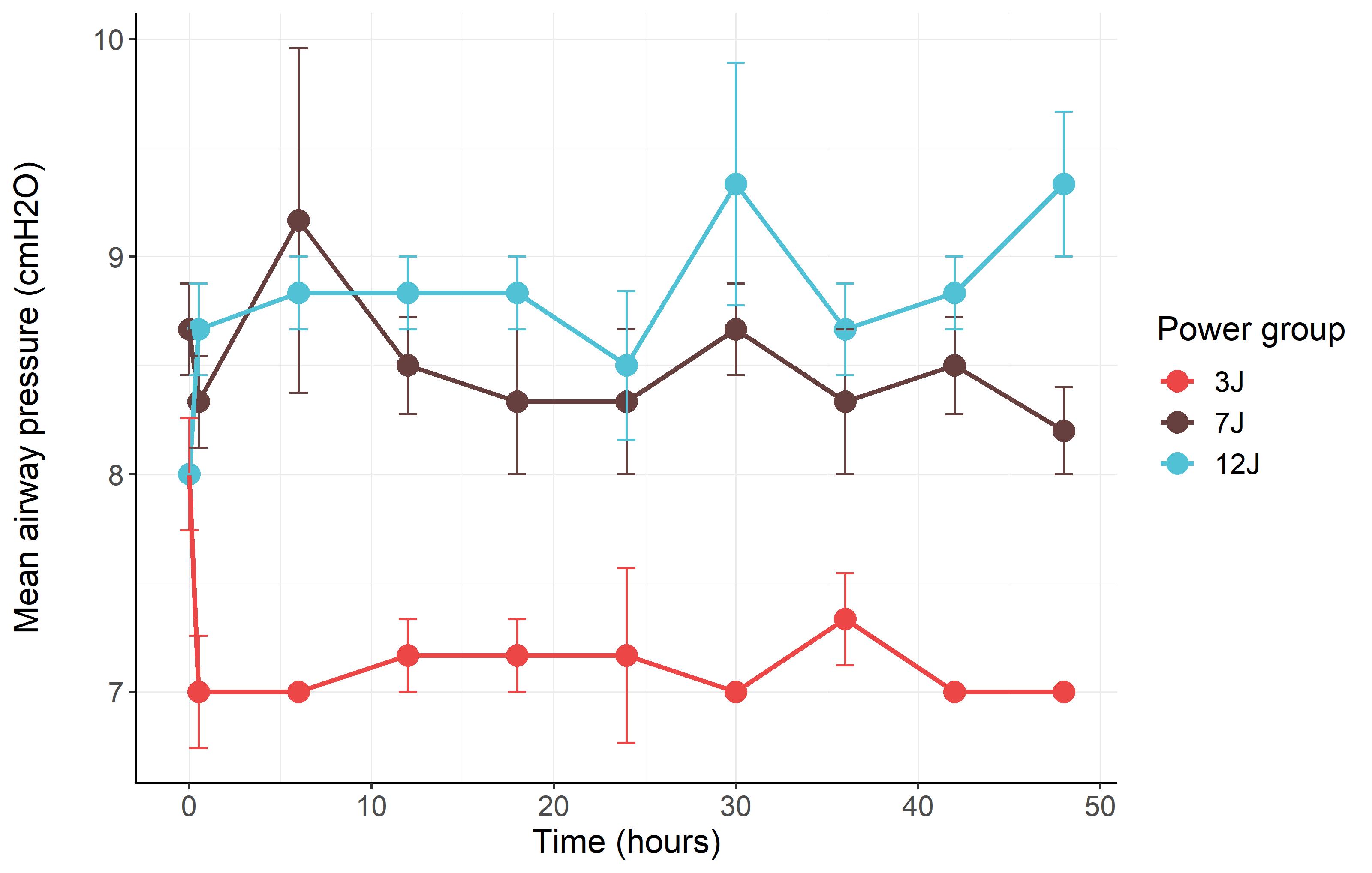


Figure E2: Mean airway pressure as a function of time in groups 3J, 7J and 12J (p values: time = 0.93, power group = < 0.001, time and power group interaction = 0.004).


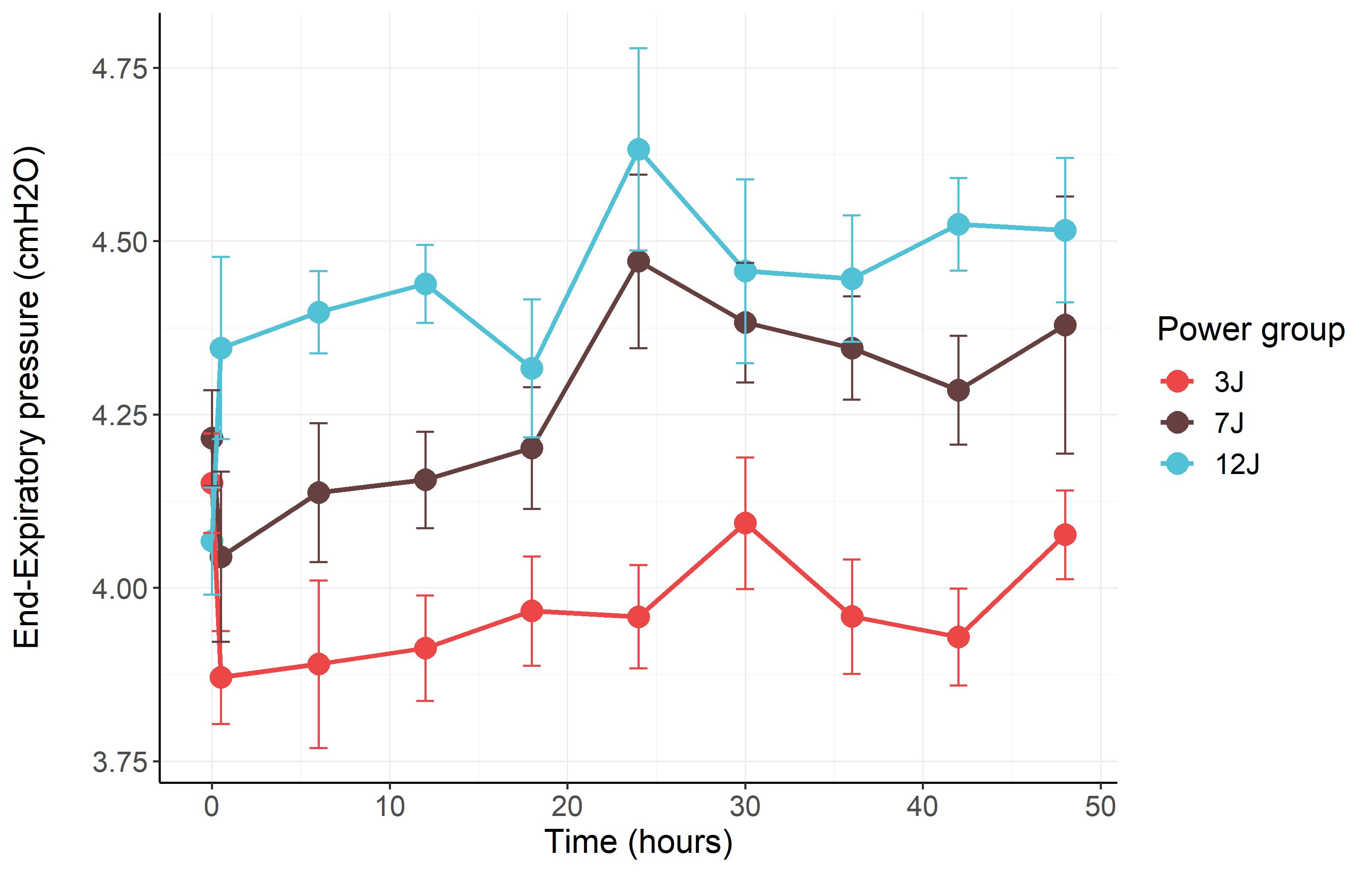


Figure E3: End-expiratory pressure as a function of time in groups 3J, 7J and 12J (p values: time = < 0.001, power group = 0.019, time and power group interaction = 0.043).


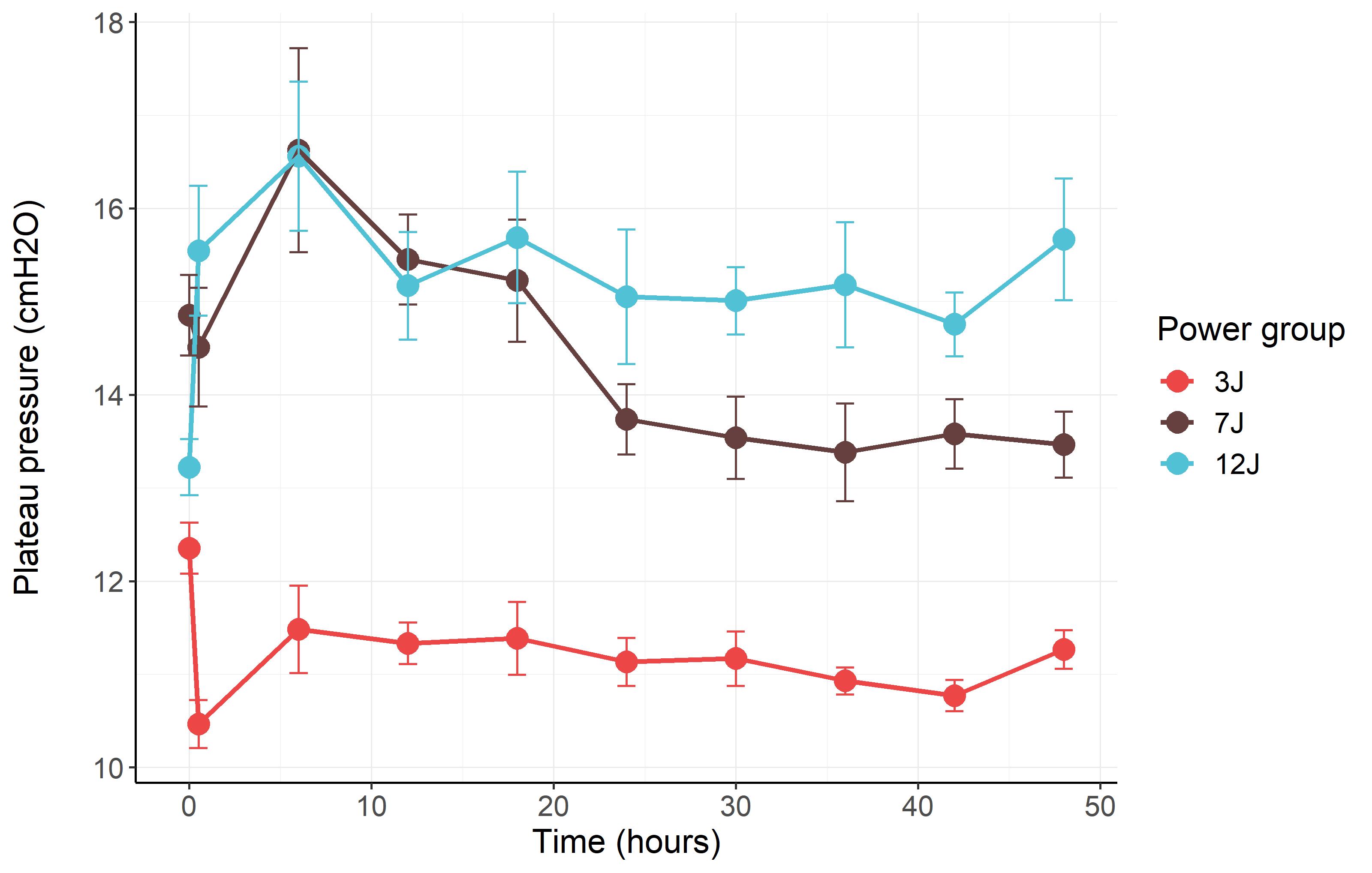


Figure E4: Plateau pressure as a function of time in groups 3J, 7J and 12J (p values: time = < 0.001, power group = < 0.001, time and power group interaction = < 0.001).


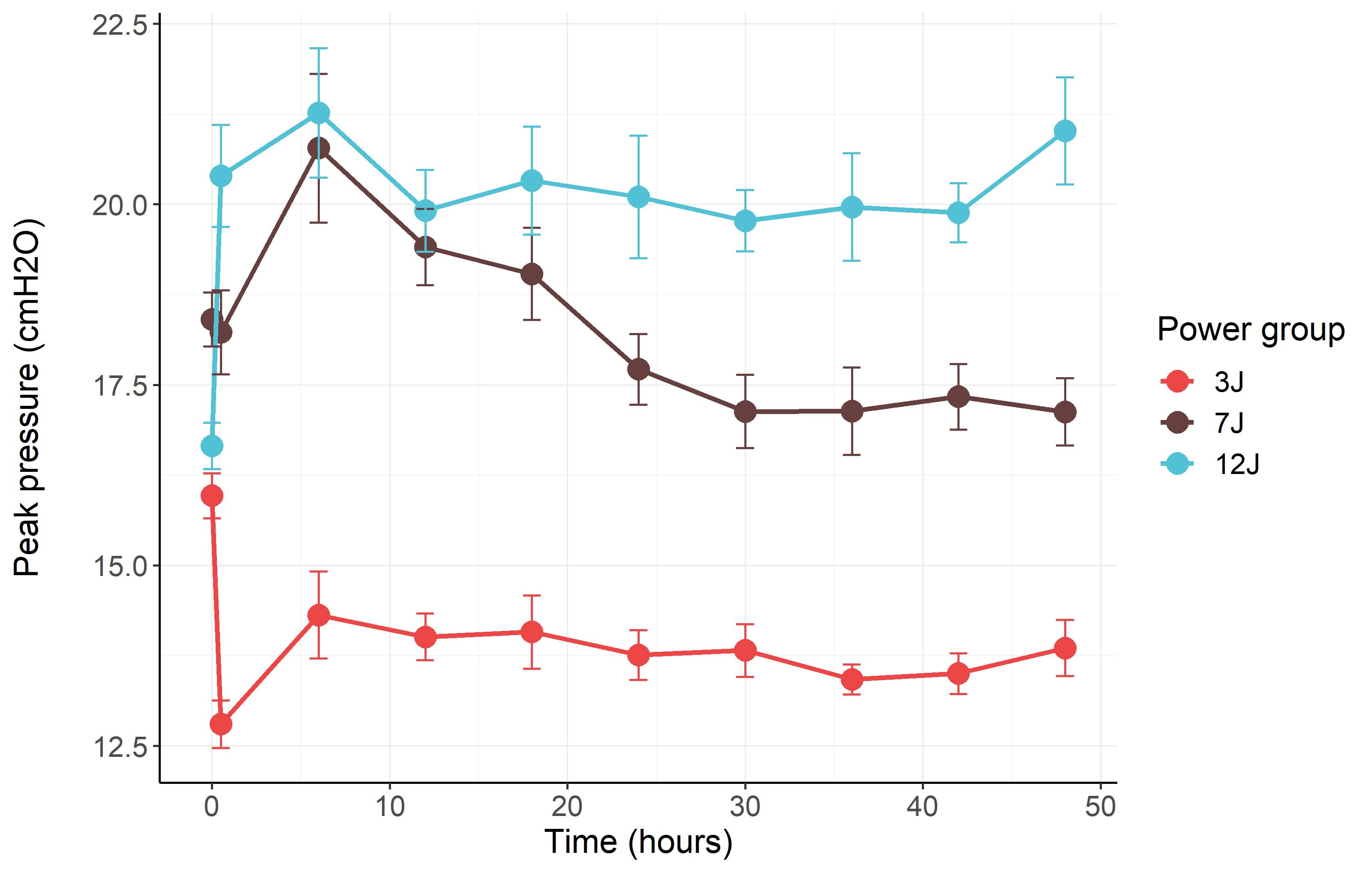


Figure E5: Peak pressure as a function of time in groups 3J, 7J and 12J (p values: time = < 0.001, power group = < 0.001, time and power group interaction = < 0.001).


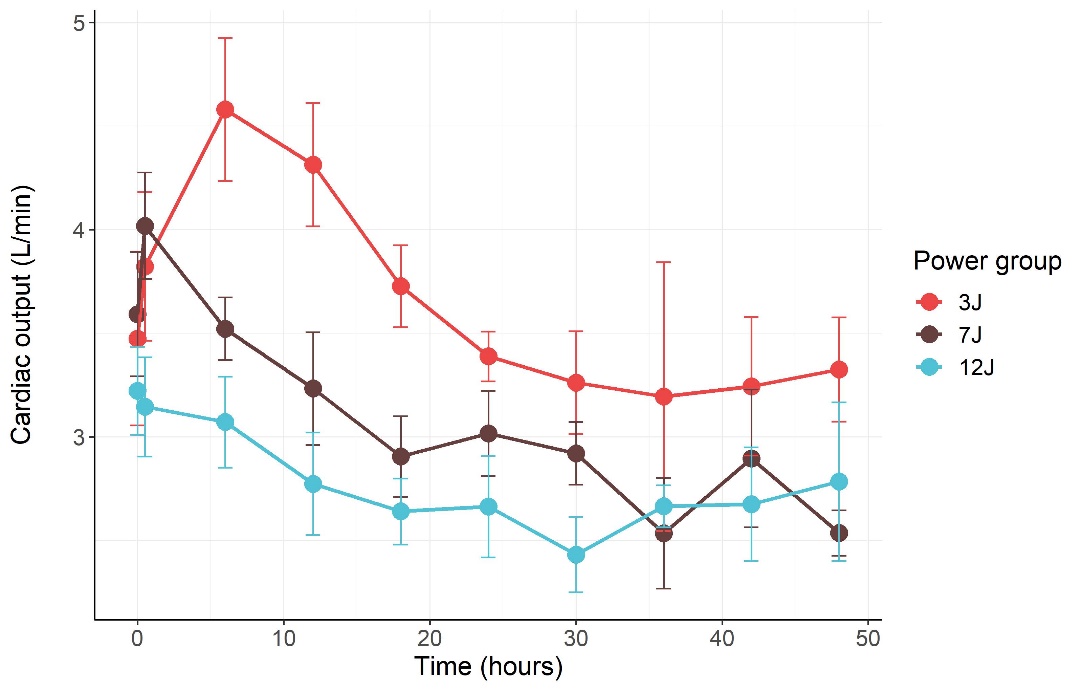


Figure E6: Cardiac output as a function of time in groups 3J, 7J and 12J (p values: time = < 0.001, power group = 0.002, time and power group interaction = 0.087).


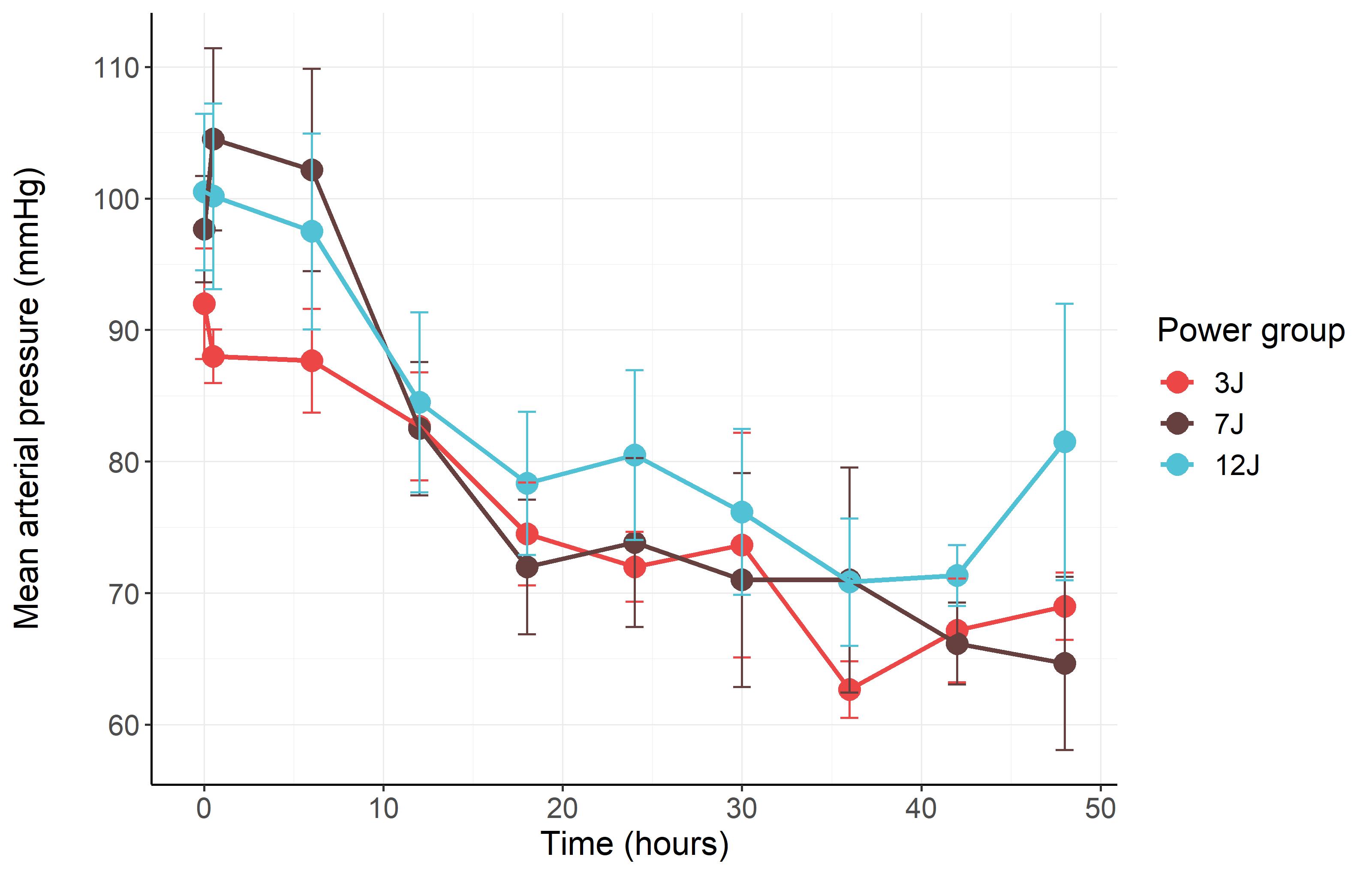


Figure E7: Mean arterial pressure as a function of time in groups 3J, 7J and 12J (p values: time = < 0.001, power group = 0.23, time and power group interaction = 0.09)


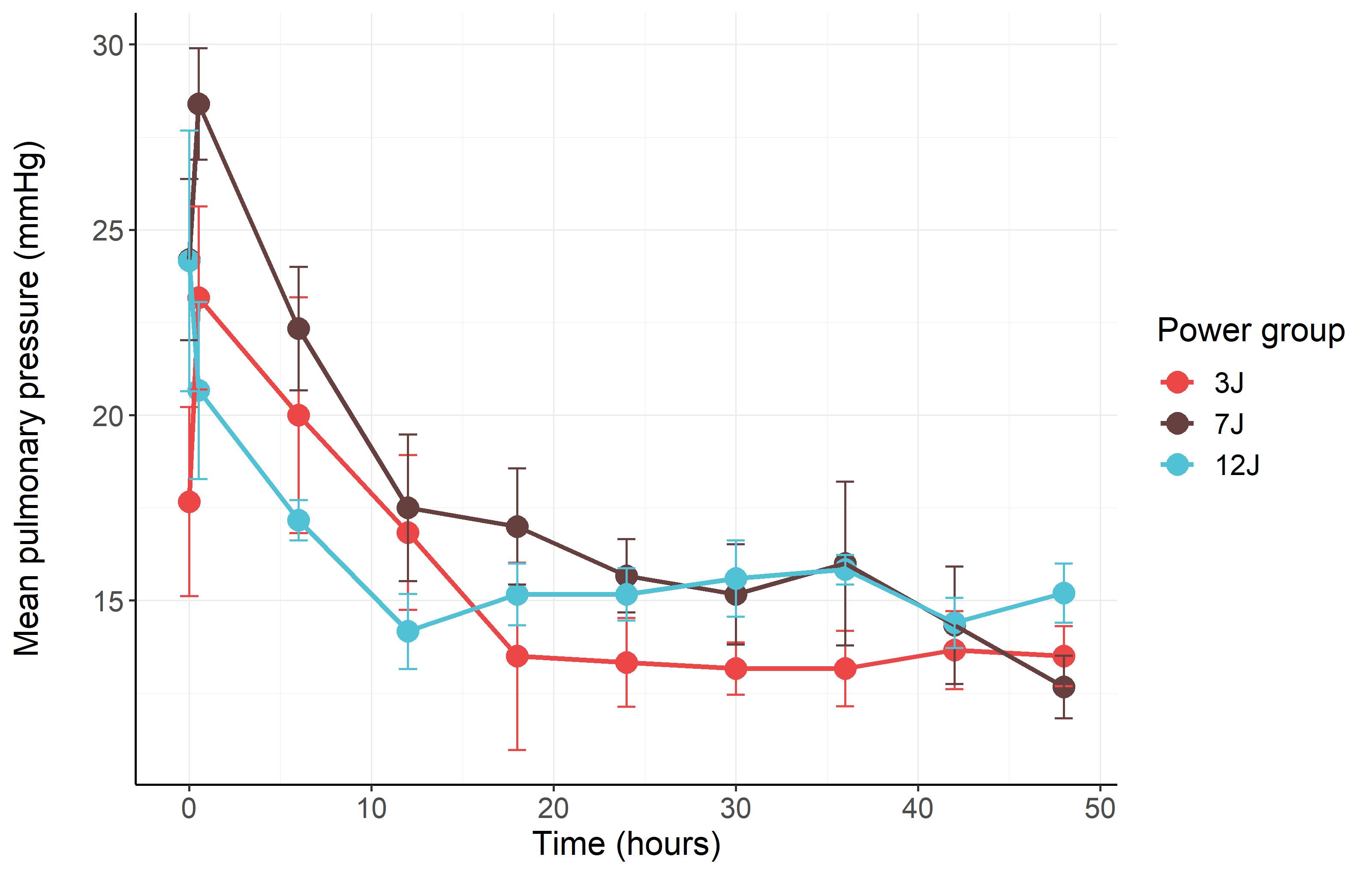


Figure E8: Mean pulmonary pressure as a function of time in groups 3J, 7J and 12J (p values: time = < 0.001, power group = 0.03, time and power group interaction = 0.01).


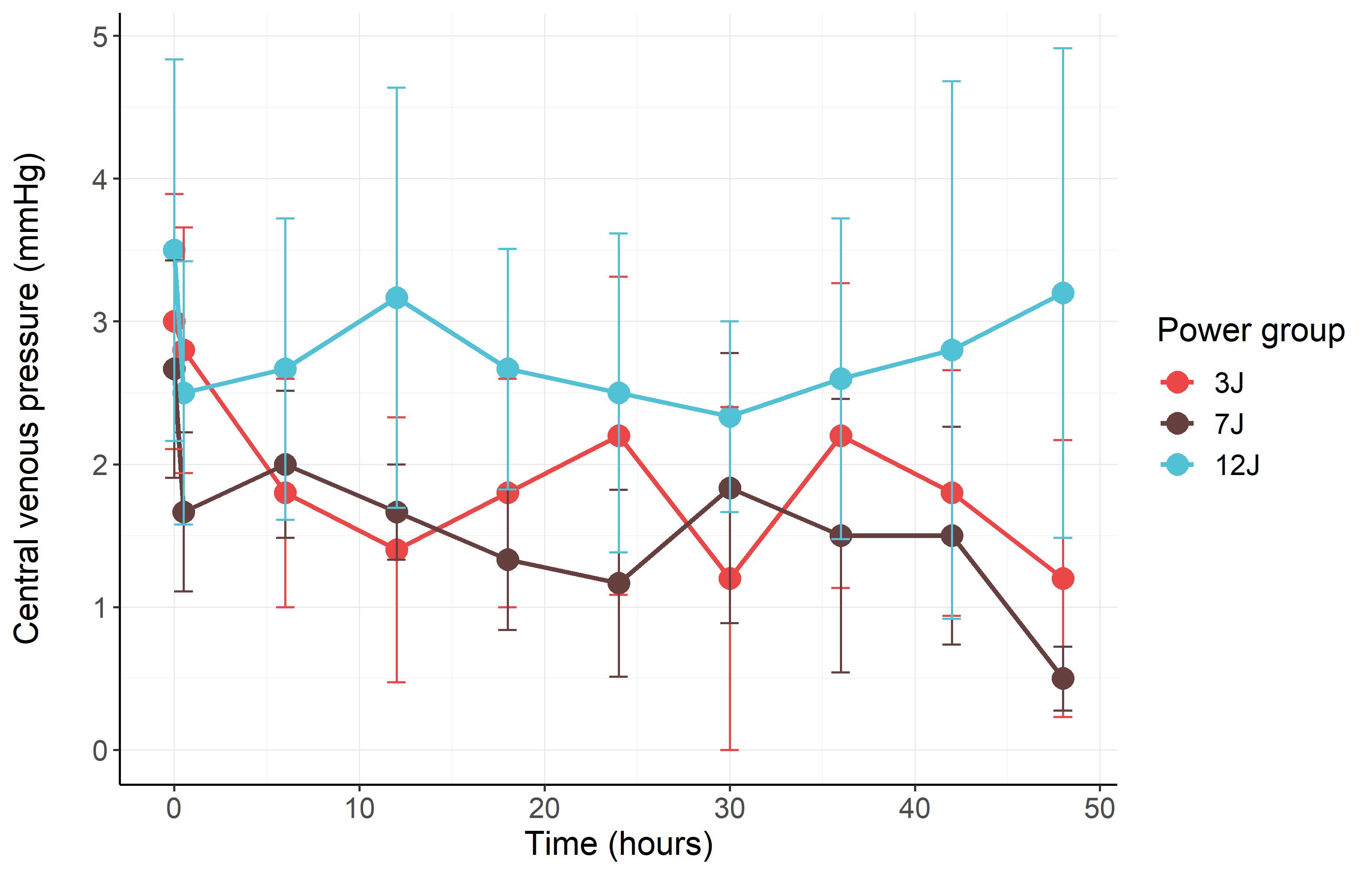


Figure E9: Central venous pressure as a function of time in groups 3J, 7J and 12J (p values: time = 0.1, power group = 0.83, time and power group interaction = 0.12).


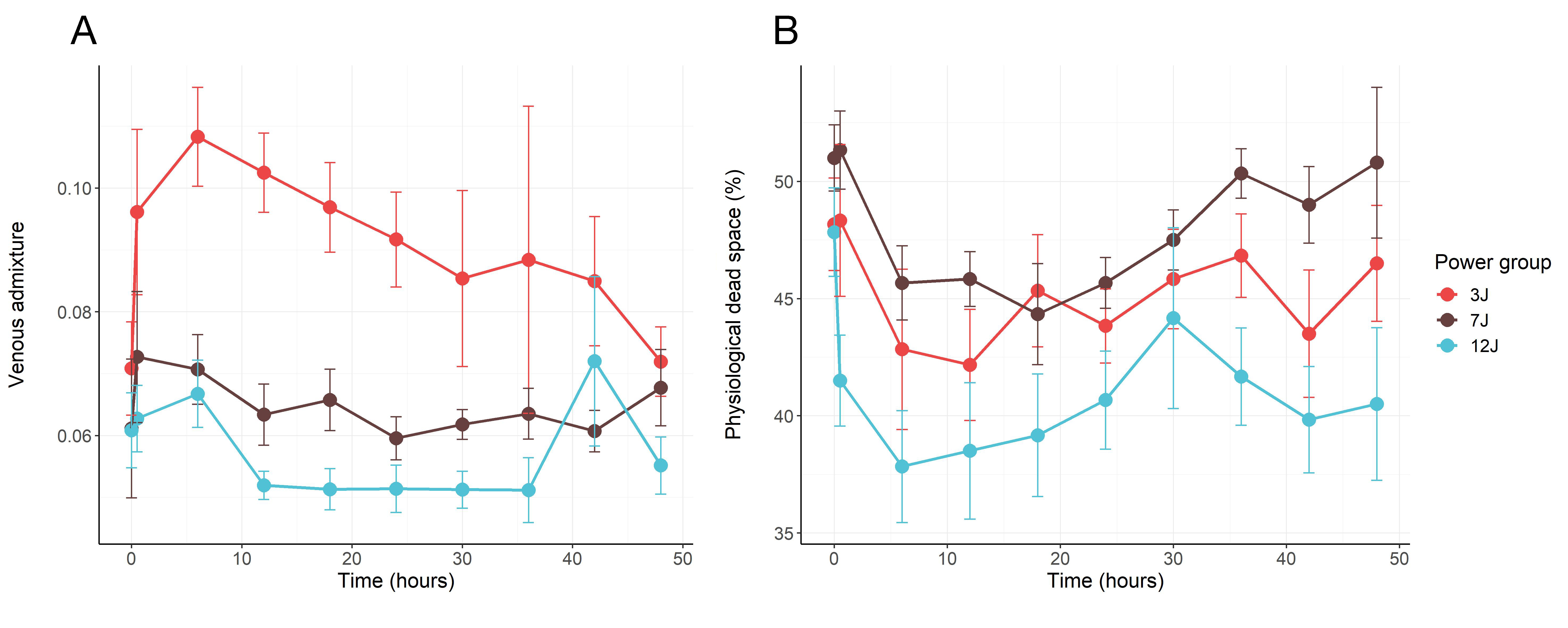


Figure E10: Venous admixture (panel A) and physiological dead space (panel B) as a function of time in groups 3J, 7J and 12J (venous admixture p values: time = 0.12, power group = < 0.001, time and power group interaction = 0.39; physiological dead space p values: time = 0.63, power group = 0.09, time and power group interaction = 0.66)


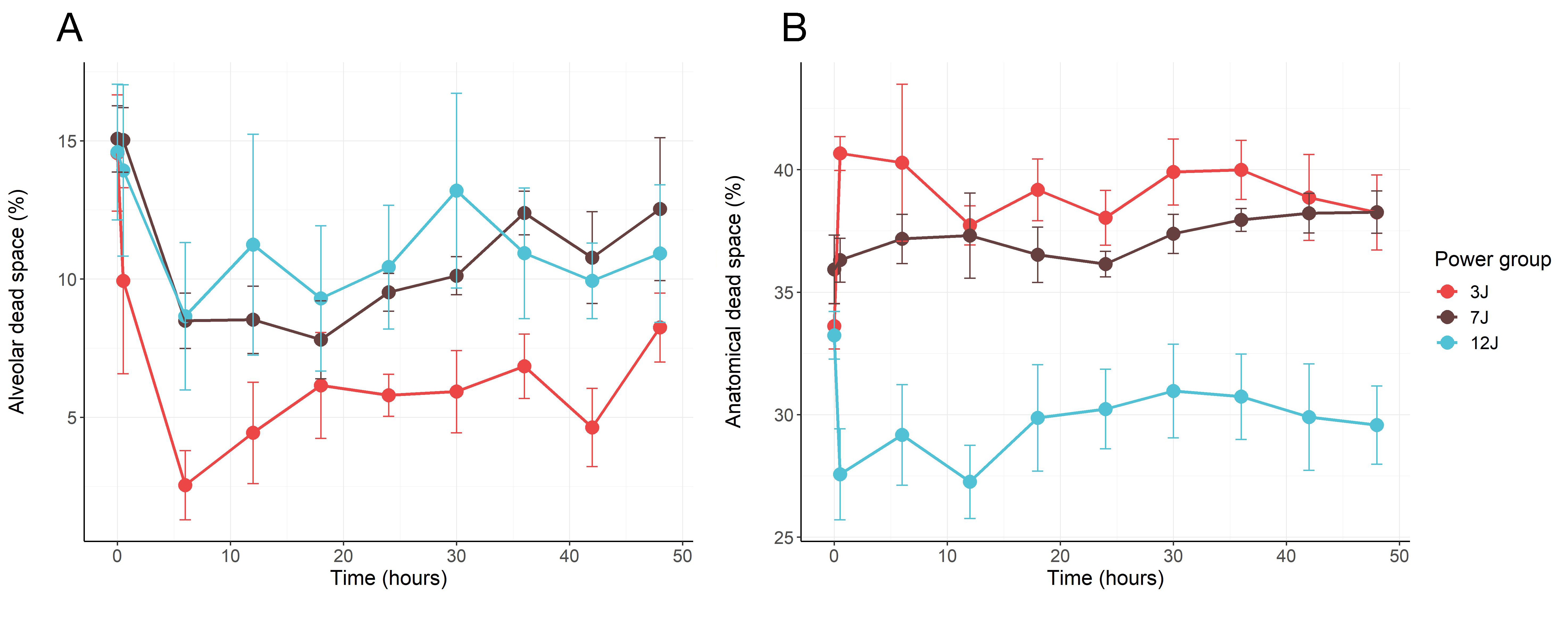


Figure E11: Alveolar (panel A) and anatomical dead space fraction (panel B) as a function of time in groups 3J, 7J and 12J (alveolar dead space p values: time = 0.04, power group = 0.02, time and power group interaction = 0.77; anatomical dead space p values: time = 0.04, power group = < 0.001, time and power group interaction = 0.65)


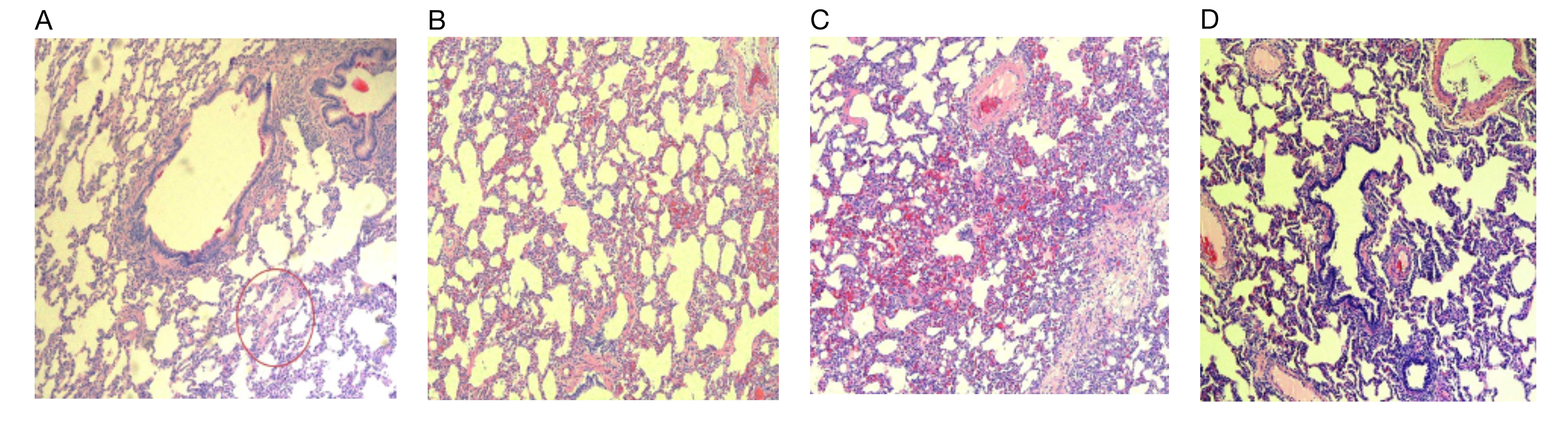


Figure E12: Example microphotographs from a control animal (panel A), 3J (panel B), 7J (panel C) and 12J (panel D). All pictures acquired at 40x.

Panel A: Area of well-preserved lung parenchyma with minimal vascular engorgement of a medium sized artery (red circle).

Panel B: Diffuse septal blood congestion with sparing of alveolar spaces

Panel C: Histological field including a medium size artery on the left and surrounding lung parenchyma characterized by severe congestion and alveolar migration of red blood cells

Panel D: Alveolar spaces preserved with evidence of congested arteries


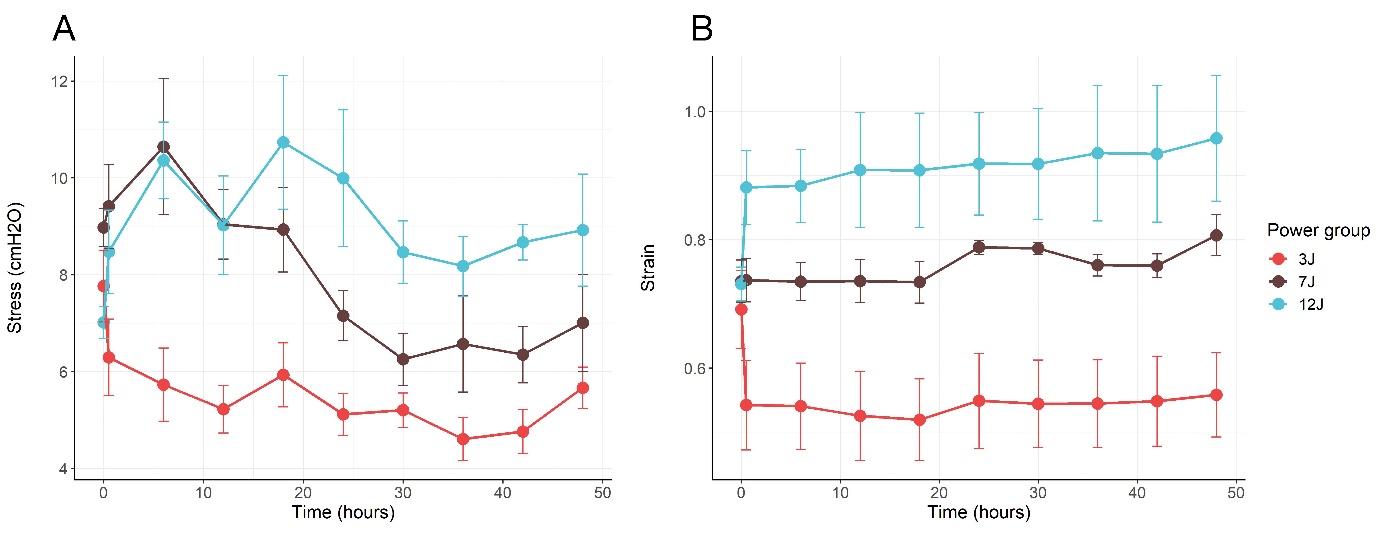


Figure E13: Stress (panel A) and strain (panel B) as a function of time in groups 3J, 7J and 12J (p values for stress: time = <0.001, power group = 0.02, time and power group interaction: < 0.001; strain: time = <0.001, power group = 0.02, time and power group interaction: < 0.001).


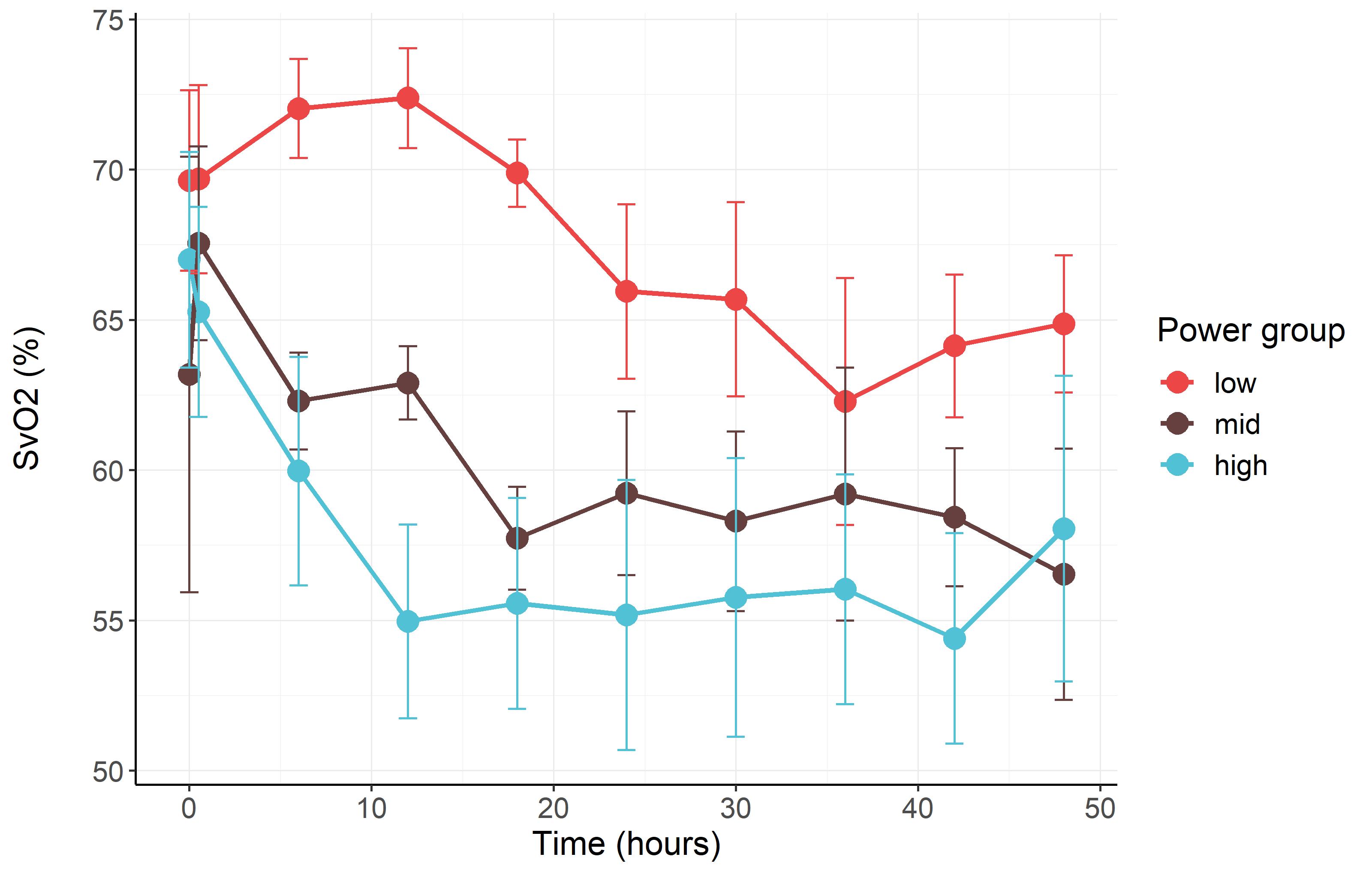


Figure E14: Mixed venous hemoglobin oxygen saturation as a function of time in groups 3J, 7J and 12J (p values: time = < 0.001, power group = 0.06, time and power group interaction = 0.95).


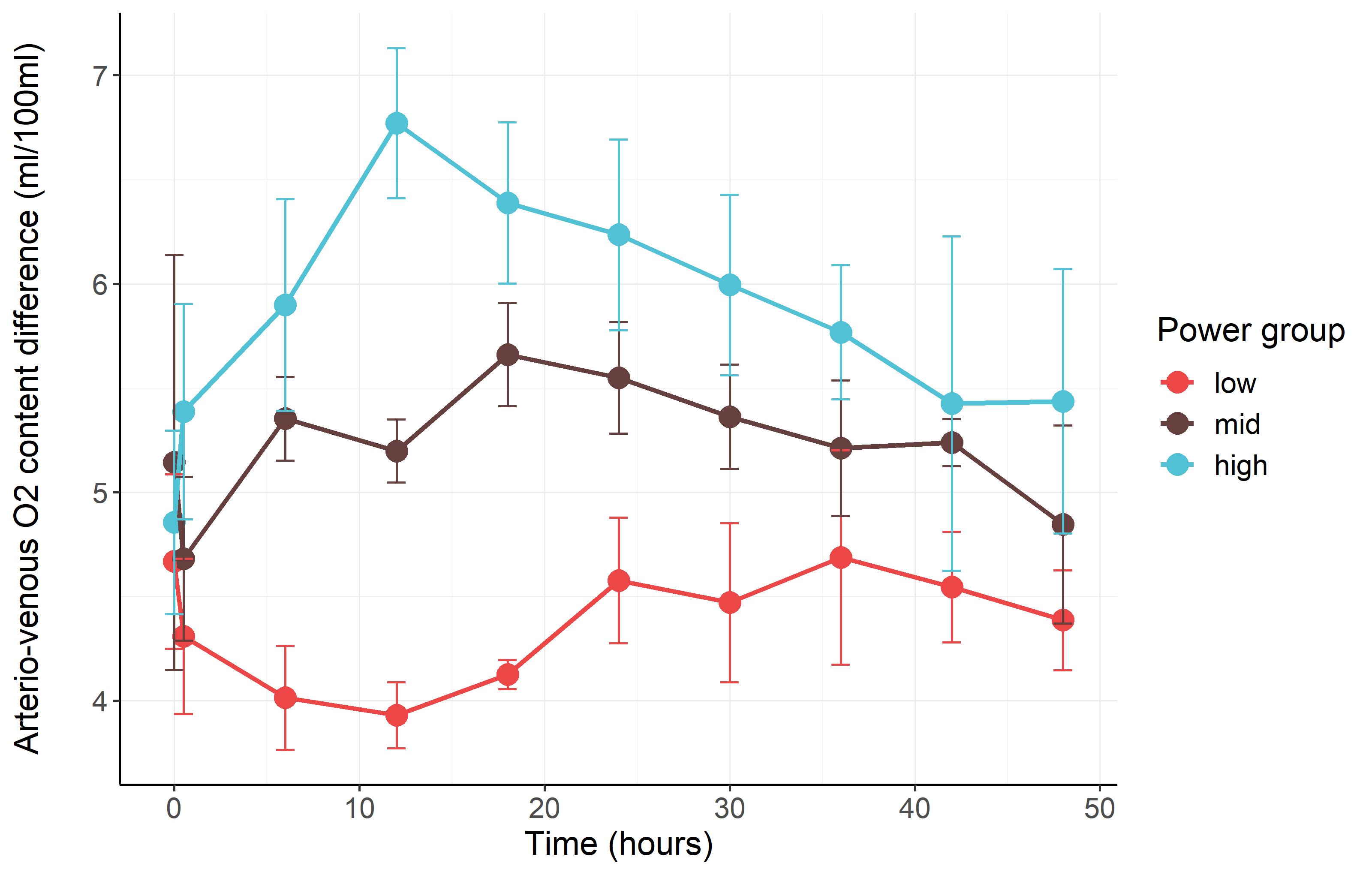


Figure E15: Arterio-venous O_2_ content difference as a function of time in groups 3J, 7J and 12J (p values: time = 0.51, power group = 0.002, time and power group interaction = 0.86).
